# Phlegmasia cerulea dolens: case report on a HIV-AIDS patient in a sub-saharian semi-urban practice

**DOI:** 10.11604/pamj.2014.19.282.4385

**Published:** 2014-11-15

**Authors:** Joseph Pierre Abah, Alain Menanga, Laah Njoyo, Josephine Ze Minkande

**Affiliations:** 1 Internal Medecine Unit, Bamenda Military Hospital, Bamenda, Cameroon; 2 Department of Internal Medicine and Specialities, Faculty of Medicine and Biomedical Sciences, University of Yaounde 1, Yaounde, Cameroon; 3 Radiology unit Bamenda Regional Hospital, Bamenda, Cameroon; 4 Department of Anaesthesiology/Reanimation, Faculty of Medicine and Biomedical Sciences, University of Yaounde 1, Yaounde, Cameroon

**Keywords:** Venous thromboembolism, phlegmasia dolens, anticoagulation, HIV-AIDS

## Abstract

Venous thromboembolism has also become a major health concern in sub-saharian Africa. Studies addressing at this issue are rare in Cameroon. Thus, the case reported here presents singular characteristics: its clinical form, phlegmasia cerulea dolens, a severe but uncommon complication of venous thromboembolism; and its infrequent recorded triggering factor, HIV-AIDS.

## Introduction

Venous thromboembolism (VTE) represents a spectrum of affections including deep vein thrombosis (DVT), pulmonary embolism (PE) and post-phlebitic disease (PPD) [1]. If PE is undoubtfully the most dangerous complication of VTE, contributing to about 15% of all hospital deaths [2], less frequent complications comprise phlegmasia alba dolens (PAD) and phlegmasia cerulea dolens (PCD). All two are severe forms of VTE resulting from acute massive thrombosis and obstruction of venous drainage of the extremity. In PCD, venous occlusion extends to collateral veins, resulting in massive edema simulating arterial embolism; that differs to PAD (an early stage of PCD) in which ischemia is not described [3]. When capillaries are involved, irreversible gangrene installs in the skin, subcutaneous tissue or the muscle. Of the reported triggering factors, cancer is the most common; others include hypercoagulation syndrome, trauma, surgery, chronic colitis, heart failure, mitral valve stenosis, vena caval filter insertion and May-Thurner syndrome. Pregnancy is another risk condition, especially during the third trimester when the uterus compresses more the left common iliac vein. We have not found a literature on PCD describing implication of HIV-AIDS, even though it is now well-established that the pandemic constitutes an emerging VTE risk factor.

## Patient and observation

**Figure 1:**
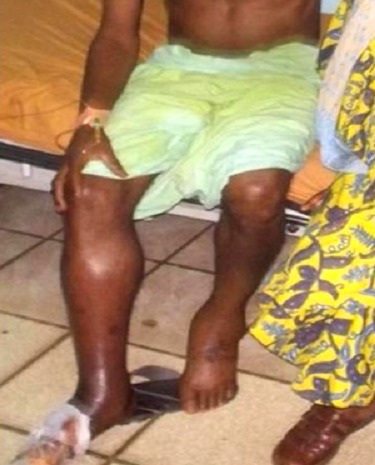
Massive thrombosis on the right lower limb: shining, swollen and discolored foot, ankle and calf with gangrenous on the foreleg

It concerns a 47 years male who was admitted at Bamenda Military Hospital. In the past history, he declared smoking (about 10 packet-years) and alcohol consumption; but he has always been well in the exception of occasional fatigue attributed to hard work. Two months previous the hospitalization, he felt pains on the right ankle; the joint also showed edema which extended upward to the knee. For the patient and his family, these manifestations were due to witchcraft; he was then treated by a traditional practitioner. As the situation worsened, with pains and edema progressing, he was forced to consult in our service. At presentation, general signs showed a temperature of 38°4 C, weight 67 kg (BMI 23.4 kg/m^2^), pulse 102, and respiratory rate 18. He was unable to walk; presenting a warm shining, painful, discolored and swollen right calf and leg. A gangrenous area was formed on the foreleg (Figure 1). The pain was more severe on pressure over the dilated and hardened popliteal and femoral vessels. Peripheral pulses were non-palpable. There was no history of trauma. This presentation was very suggestive of phlegmasia cerulea dolens.

### Investigations

**Figure 2:**
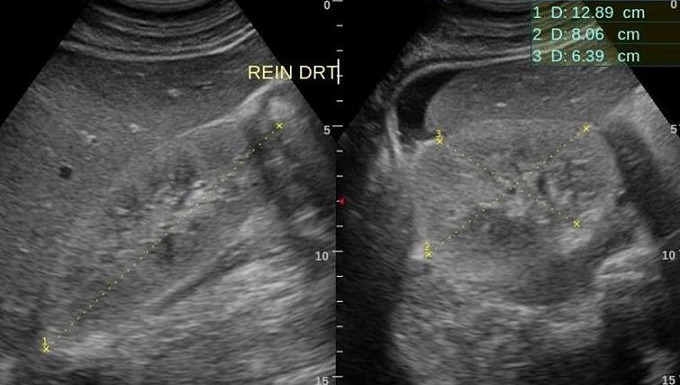
Ultrasound of the right kidney showing characteristic lesions of HIV-related nephropathy (HIVAN): increased size, maintained cortico-medullary differentiation and marked echogenicity

ESR 120 mm/first hour; CRP 48 mg/L; blood urea 0.65 g/L; creatininemia 17 mg/L; HIV positive; CD4 count 209 cells/µL; syphilis and chlamydia serologies negatives; aspartate amino-transferase 45 UI/L; alamine amino-transferase 37 UI/L; Gamma-glutamyl transferase 49 UI/L; alkaline phosphatase 65 UI/L; albuminemia 35 g/L; TP 89%; INR 1.2; Urine stick normal; fasting blood sugar 1.65 g/L. -EKG: sinusal tachycardia (105/bpm). Chest X-ray: normal heart shadow; clear lung parenchyma. -Lower limb ultrasound: complete thrombosis in the right limb, extended from the tibial veins, popliteal and to the femoral veins; and very weak pulse in the arteries. -Abdominal echography: normal sized kidneys with bilateral increase in echogenicity (exceeding liver parenchymal) and a maintained cortico-medullary differentiation (Figure 2). The retained diagnosis was phlegmasia cerulea dolens. The patient received intravenous fluids, subcutaneous low-molecular-weight heparin and oral anticoagulant with monitoring of INR. His right leg kept elevated. Treatment also included mixt short and long acting insulin, antibiotics, antiretroviral drugs (HAART), sub-cutaneous morphine. If fever ceased, swelling and discoloration progressed upward to mid-thigh. The patient was transferred to a vascular surgery center where an amputation was made.

## Discussion

Phlegmasia dolens (PD) is a rare complication of VTE. It occurs at all age but is more common in the fifth and sixth decades [2]. Gender predominance is not established; left-sided involvement is more frequent (3 to 4: 1 ratio). Upper limbs are involved in less than 5% [2]. PD results of acute and massive thrombosis in the lower limb veins with significant compromised vein outflow. It comprised phlegmasia alba dolens (PAD), phlegmasia cerulea dolens (PCD) and venous gangrene. Its pathogenesis is unclear. Deep vein channels are involved by an extended thrombosis. Hydrostatic pressure exceeds oncotic pressure, and it leads to a huge interstitial fluid sequestration and edema. Fluid sequestration can reach 5-10 liters within days. Circulatory shock may result in about 1/3 of patients, and arterial insufficiency may follow [2]. In PAD, thrombosis does not extend to collateral veins, edema is less important and there is no ischemia, contrarily to PCA where congestion is more important. Gangrene occurs when thrombosis involves capillaries; lesions have then become irreversible, conducting to limb necrosis. Venous gangrene differs of arterial as the necrosis installs despite palpable or Doppler identifiable peripheral pulses. Malignancy is the most common risk factor (present in 20-40% of patients with PD) ^3^. Other associated risk factors include hypercoagulable syndrome, surgery, trauma, chronic colitis, gastroenteritis, heart failure, mitral valve stenosis, vena caval filter insertion, May-Thurner syndrome (compression of the left iliac vein by the right iliac artery), and pregnancy (at the third trimester). Although HIV-AIDS is now well known as an important VTE risk factor, the pathogenesis, not clearly described for VTE, is more obscure for phlegmasia dolens. HIV infections as itself associated to the opportunistic diseases are likely very thrombogenic. Some studies have identify factors related to HIV infection, and demonstrated a strongest association with VTE such as, low CD4 (+) cell count, protein S deficiency, protein C deficiency and circulating antibodies (antiphospholipid, anticardiolipid, etc...)[4]. As the same, diabetes is not known as a set off factor for PD, even though one case reported in India concerned a diabetic patient [3]. Literature does not mention whether atherosclerosis (potentially induced, in our case, by smoking and diabetes), can play a role in the onset of PD by pre-fragilizing arteries.

In the end, HIV-AIDS was the main VTE risk factors present in our patient. He did not have any of the reported triggering factors of PD. Clinically, PD presents as a triad of edema, agonizing pain and cyanosis. Conservative treatment of PD concerns PAD and mild PCD, and includes steep leg elevation, anticoagulant and fluid resuscitation. It aims to prevent venous gangrene. Steep leg elevation helps to decrease edema, while anticoagulant purposes to decrease proximal clot propagation. Paquet proposed to use thrombolysis for the treatment of PCD [5]. Other management approaches consist of catheter-directed thrombolysis associated to high doses of urokinase or tissue plasminogen activator (t-PA), or intra-arterial low-dose thrombolysis. The latest seems to be more efficient when venous gangrene is installing as the agent is delivered to arterial capillaries and, then after, to venules. Surgical thrombectomy allows rapid decrease of the hydrostatic pressure. Fasciotomy alone or associated to thrombectomy, or thrombolysis reduces compartimental pressure; though, it significantly increases morbidity because of the prolonged wound healing and the risk of infection. Conservative management is associated to a high incidence of post-phlebitic syndrome (94% among survivors) [2]. Despite all the therapeutic modalities mentioned above, PCD and venous gangrene still remain life-threatening and limb-threatening conditions with a very high mortality (20-40%) [2]. Pulmonary embolism is responsible of 30% of the deaths related to PCD. Incidence of amputation attains 12-50% among survivors.

## Conclusion

This case of PD appears important to be made aware with regards of its association with the HIV pandemia, and the history of its management out lighting the confrontation that still exist in subsaharian Africa between biomedical knowledge and socio-cultural believes.
